# The Habituation/Cross-Habituation Test Revisited: Guidance from Sniffing and Video Tracking

**DOI:** 10.1155/2016/9131284

**Published:** 2016-07-19

**Authors:** G. Coronas-Samano, A. V. Ivanova, J. V. Verhagen

**Affiliations:** ^1^The John B. Pierce Laboratory, 209 Congress Avenue, New Haven, CT 06519, USA; ^2^Department of Surgery, Yale University School of Medicine, New Haven, CT 06520, USA; ^3^Department of Neuroscience, Yale University School of Medicine, New Haven, CT 06520, USA

## Abstract

The habituation/cross-habituation test (HaXha) is a spontaneous odor discrimination task that has been used for many decades to evaluate olfactory function in animals. Animals are presented repeatedly with the same odorant after which a new odorant is introduced. The time the animal explores the odor object is measured. An animal is considered to cross-habituate during the novel stimulus trial when the exploration time is higher than the prior trial and indicates the degree of olfactory patency. On the other hand, habituation across the repeated trials involves decreased exploration time and is related to memory patency, especially at long intervals. Classically exploration is timed using a stopwatch when the animal is within 2 cm of the object and aimed toward it. These criteria are intuitive, but it is unclear how they relate to olfactory exploration, that is, sniffing. We used video tracking combined with plethysmography to improve accuracy, avoid observer bias, and propose more robust criteria for exploratory scoring when sniff measures are not available. We also demonstrate that sniff rate combined with proximity is the most direct measure of odorant exploration and provide a robust and sensitive criterion.

## 1. Introduction

The olfactory habituation/cross-habituation test (HaXha) is a noninvasive spontaneous behavioral task that has been used to study the ability to smell and the capacity to discriminate between stimuli (odors) in a large variety of animals and humans. The HaXha follows the basic principles shown by Thompson and Spencer in 1966: when any stimulus is repeatedly evoked, the behavioral response decreases (habituation), not involving sensory adaptation/sensory fatigue or motor fatigue. Meanwhile the presentation of a different stimulus leads to a change in the amplitude of the habituated response (cross-habituation) [1, 2].

The general protocol for olfactory HaXha in rodents consists of presenting an odor (in a paper filter or cotton applicator) generally in the center of the experimental cage and measuring the time in which an animal is oriented to and within 2 cm of the odorant. To measure the habituation phase, the novel stimulus is presented several times (trials). The cross-habituation phase can be studied by changing the stimulus for a novel unfamiliar odor. This protocol has been adapted to different species, for example, by increasing the number of trials in rats [3], or by increased intertrial intervals in guinea pigs [4]. The first HaXha experiments in rats demonstrated that male and female rodents are able to discriminate between different urine odors independently of hormone status [5] and later this behavioral task was adapted to mice using sex related odors as stimuli [6].

Olfactory habituation can be mediated by different circuits of the olfactory system when using paradigms that differ in timescale. Short-term habituation following 20 sec odor presentations and short intervals (10 sec) is mediated by neuronal adaptation lasting about 2 minutes in the anterior piriform cortex [7]. Long-term habituation [8–10] following odor presentations of 50 sec separated by 5 min intertrial intervals persists up to 30 min and is mediated by the olfactory bulb.

To study olfactory alterations researchers have also used other behavioral olfactory-guided tasks such as the social transmission of food preference, which combines social interactions and olfaction [11], the odor-cup sand-digging task, in which the animals have to be trained to dig into one of two cups (S+, S−) [12], the buried food test, which tests the ability of the animals to smell a piece of familiar food such as cereal hidden under the bedding of the cage [13]. In all of these tasks the animals have to be food-regulated in order to obtain responses. For the odor-cued taste avoidance [14], which combines odor detection and odor discrimination, the mice have to be thirsty to perform the task. The large advantage of the HaXha task is that animals do not need to be food- or water-regulated to perform the task. This is critical for aging research where food restriction may interfere with the aging process, for example, by affecting metabolic rate or inducing stress [15]. Another advantage is that the HaXha test is a spontaneous odor discrimination task which requires no training which may involve cognitive processes unrelated to the ones being under investigation.

Despite these advantages and ability to target the patency of different neural structures by adjusting the timescale, the standard means to measure the exploration time is by a human experimenter using a stopwatch. This provides the potential for observer bias in cases where subjects cannot be tested without prior knowledge of status, as when testing anatomically different phenotypes (e.g., fur, body weight). Further, as individual exploratory bouts can be quite short (<1 sec) the use of a stopwatch limits accuracy. Last, a human observer will have limited accuracy in scoring the behavior according to exact criteria such as proximity within 2 cm of the odor source and precise head angle toward it.

To avoid these potential confounds altogether we used behavioral video tracking software (Noldus), which is able to recognize the nose, the center, and the tail of the mouse at 10 frames/sec. This allowed us to quantify the distance between the nose and the odor stimulus, the head angle to the odorant, and the locomotion of the animal (position and velocity). Noldus also automates trial start and end.

While it seems to be taken for granted that animals are “smelling” the target odor while the standard criteria of proximity and orientation are met, odor exploration fundamentally involves sniffing, the rate of which is actively modulated, increasing when rodents explore novel odors [16]. Therefore, the assessment of odor-guided (dis-)habituation should also be guided by sniff rates, which standard HaXha tests do not include but which we also measured here using whole-body plethysmography.

Sniffing is characterized by a rhythmic inhalation and exhalation of air through the nose. This behavior also plays a critical role in shaping how odor information is represented and processed by the nervous system. Sniffing behavior in rodents is dynamic, varies with the behavioral context, and is modulated by olfactory and nonolfactory processes [17]. Meanwhile, the frequency of sniffing (sniff rate) has been used as a parameter to characterize sniffing behaviors in rodents, as they increase from 2 Hz to 4–12 Hz when they are investigating novel odors [18, 19].

Identification of olfactory dysfunction at early stages of sAD has been proposed as a promising diagnostic tool, potentially allowing early treatment before the irreversible cognitive deteriorations are established. However, there are many issues in this field to be resolved, including finding optimal behavioral tasks to aid diagnosis. Averback described neurodegeneration in the anterior olfactory nucleus in the olfactory bulbs and tracts, caused by cells loss in the presence of amyloid plaques and neurodegenerative tangles [20]. Posthumous histological analysis suggested a relationship between the olfactory sensory pathway and the severity of the disease [21, 22]. Warner et al. first suggested olfactory impairments in patients with AD, employing a standardized smell identification test [23]. However, the lowered ability to distinguish between or recognize different odors is not exclusive for AD, as olfactory dysfunctions have also been detected in Parkinson's disease [24, 25], multiple sclerosis [26, 27], viral infections [28, 29], lesions in the brain [30], and aging [31]. Thus, while there is a need for an olfactory test for AD, it should also be specific.

Recently, olfactory alterations in transgenic mice models for familial AD were detected with HaXha using food and sex-related odors as the stimuli, where olfactory deficits were correlated with the stage of the disease [32, 33]. Our group studies the olfactory behavior of Fus1 KO mice, a novel model of accelerated aging and sporadic Alzheimer's Disease (SAD), in which deletion of a mitochondrial tumor suppressor protein Fus1/Tus2 leads to an overproduction of reactive oxygen species (ROS) inducing oxidative damage to cellular macromolecules [34, 35]. Our previous studies suggest that the female Fus1 KO mice at 10 months old have alterations in their habituation to nonsocial odors and deteriorations in their cross-habituation for social odors (ms submitted for publication).

Here, we propose a novel approach to accurately assess HaXha using the Noldus system to track the behavior of every animal combined with whole-body plethysmography to noninvasively evaluate their sniffing. We evaluated how the classical HaXha odor exploration criteria relate to sniffing in both wild type and FUS1 KO mice. We further sought to find improved nonolfactory parameters, to be used in cases when sniffing cannot be measured. We confirm that standard criteria accurately capture sniffing-mediated olfactory exploration and suggest that velocity combined with a relaxed distance criterion outperforms odor exploration quantification.

## 2. Experimental Procedures

### 2.1. Subjects

In this study we compared the behavior of young Fus1 KO/129sv and WT/129sv mice of different genders generated by Dr. Alla Ivanova [35]. The groups in the study were 4-5-month-old female Fus1 KO (FUS ko; *n* = 13) and WT mice (*n* = 7). The vivarium had a 12 h/12 h inverted light cycle with lights off at 10:30 am. All animals were housed individually in polycarbonate cages (12 × 12 × 25 cm) with controlled humidity (40%) and temperature (22°C) and were provided with nestlets.

### 2.2. Odor Habituation/Cross-Habituation Task

A cotton-tipped wood applicator (Puritan REF 806-WC) was presented mounted in a removable holder on the bottom of the cage, located 1 cm above the cage floor. We saturated the cotton applicator with one of 4 odorants: mineral oil (MO), amyl acetate (AA) 1% (in MO), phenyl ethanol (PE) 1% (in MO), and social odorant (S, obtained by swabbing the cage of a female mouse). A total of 12 trials were performed per mouse, where each odorant was presented three times in succession per daily session to yield the following order: MO1–3, AA1–3, PE1–3, and S1–3. The total duration of a single session was 35 min per mouse, each trial consisting of 2 min per odorant exposure and an intertrial interval of 1 min between stimuli. The HaXha test was performed twice (48 h between sessions). Odor exploration was defined as being oriented toward the applicator tip while the nose was within 2 cm of it. This test was adopted from previous reports [25, 32]. This test evaluates if mice are able to spontaneously recognize a novel odorant stimulus by spending more time smelling the applicator (cross-habituation phase), as opposed to the time that the mice spend on each repeated stimulus (habituation phase).

### 2.3. Video Tracking and Sniffing System

In order to reduce timing errors and experimenter bias in measuring the habituation/cross-habituation task and to know if these behavioral responses were specific to the olfactory abilities of the animals, we combined a system to automatically record behavioral responses using the Noldus behavioral tracking system (EthoVision XT, version 10.1, Noldus Information Technology B.V., Wageningen, Netherlands) with sniffing analysis obtained by whole-body plethysmography. We used an air-sealed experimental semitransparent white acrylic box (26 × 38 × 16 cm) with a USB camera (Logitech HD Pro C920, 1920 × 1080 pixels) mounted at the ceiling of the box, aimed downward. Noldus software analyzed the camera input to identify and score the behavior of the animal. In Noldus, we marked a circular area (OD = 4 cm, *A* = 12.6 cm^2^) in the middle of the cage floor where a cotton swab was placed. We saturated the cotton applicator with one of the following experimental odors: MO, AA, PE, and S.

To measure sniffing noninvasively, a pressure sensor (Buxco, TRD5700) communicated with the experimental box. The transducer signals were amplified 10^6^x (A-M systems, differential AC Amplifier model 1700), band-pass filtered between 0.1 and 40 Hz and 60 Hz notch filtered (A-M systems), and subsequently filtered between 0.1 and 40 Hz with an 8th order Linkwitz-Riley filter using a MiniDSP 2 × 4 processor (MiniDSP, Hong Kong). The data obtained from the sensor was stored using a Neuroplex system (RedShirtImaging, Decatur, GA, USA), synchronized to the start of each trial using the Noldus mini-USB i/o box.

During 2 minutes of each trial the behavior of a mouse was recorded, and when the rodent's nose was oriented to the cotton within a 2 cm distance a signal was generated via the Noldus mini-USB i/o box, identifying the period that the mouse was exploring the stimulus, and sent to the Neuroplex data-acquisition system. Noldus also scored the number of times the mouse oriented to the stimulus and the distance and speed traveled by the rodent during every trial. The data was analyzed using Noldus for behavioral responses and using MatLab for sniff rates for the period that the animal was exploring versus not exploring the stimulus.

### 2.4. Combined Video Tracking and Sniffing Analysis

Matlab (R2016A, the Mathworks, MA) was used to analyze all raw sniffing data and the Noldus “exploration” signal from Neuroplex and video data from Noldus exported in Excel format at 100 ms bins. Only the trial types PE3, S1, S2, and S3 were used, as S1 was by far the most explored stimulus. Matlab output was organized in Excel (2016 for Mac, Microsoft) for calculating means and sem (sd/√*n*) and graphing.

For each trial (120 s duration) sniffs were identified by filtering the Neuroplex data (200 samples/s) with a 4th-order Butterworth bandpass (3–20 Hz) filter and finding peaks in *z*-scored data exceeding 0.4 s.d. Sniff amplitudes were identified by finding local maxima around sniff times. Average sniff rates and amplitudes during Noldus identified exploration on/off times were calculated. Neuroplex variable acquisition delays common to its “BNC-only” mode were taken into account.

For deeper analyses the following procedure was used to integrate the Neuroplex-based sniffing signal (200 s/s) with the Neuroplex video tracking output (10 s/s). Mouse nose and core location (*x*, *y*) and velocity data with rare missing values were completed by using last-known locations and velocities, except for start values which were set to 1 (location) or 0 (velocity). Instantaneous sniff rates, velocity (cm/s), distance (cm) to odor (at *x* = 0, *y* = 0), and head angle to odor were assigned to each 100 ms bin and subsequently convolved with a 30-bin Gaussian low-pass filter and end-corrected. Odor exploration criteria were based on thresholding these smoothed signals.

First-order regressions were calculated between sniff rates and distance, velocity, and the angle to odorized cotton tip (Matlab “regression” function). Multiple regression (Matlab “fit” function) was performed of 1st and 2nd order (“poly11” and “poly22”) onto sniff rate (including nonzero constants). This was also performed for each trial type (PE3, S1, S2, and S3) and all trials combined per group (wild type (WT) or FUS KO) by combining data bins of all trials into population vectors. Stepwise multiple regression was computed using population vectors for regressing angle, distance, and velocity (Matlab “stepwiselm”) onto sniff rate on this. Population vectors were also used to parametrically explore how combinations of exploration criteria affected exploration on/off time and sniff rates. All data are shown as mean ± SEM (sd/√*n*).

## 3. Results

**Figure 1:**
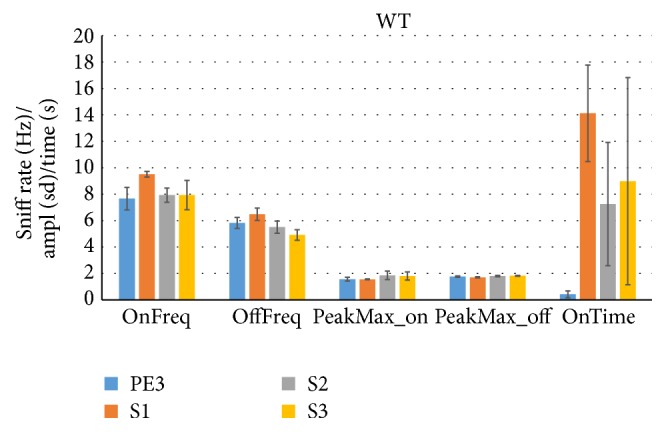
The sniff rate increases during cross-habituation in female wild type (WT) mice. A cotton tip odorized with phenyl ethanol (PE) or mouse urine as social odor (S) was presented 3 times during the habituation/cross-habituation test (HaXha). PE3 represents the 3rd presentation of PE and S1 the 1st presentation of S. The time exploring the odorant (OnTime) by WT mice (129 strain, *n* = 7) was scored in real-time by Noldus video tracking system based on the standard criteria of nose proximity (<2 cm) and head orientation (<20°) to the odor source. The simultaneously plethysmographically measured sniff rate was measuring while exploring (OnFreq) and not exploring (OffFreq), as was the sniff amplitude (PeakMax_on and PeakMax_off, resp.). Increased exploration time during S1 shows cross-habituation and was accompanied by an increase in the sniff rate. OnFreq and OffFreq: sniff rate (Hz); PeakMax_on and PeakMax_off: sniff peak amplitude (standard deviations of *z*-scored pressure signal). OnTime: odor object exploration time (s). All data are mean ± SEM.

The olfactory HaXha task is used to evaluate the patency of the olfactory system by asking how much time an animal spontaneously explores new or previously presented odor objects. It is hence assumed that the exploration time is equivalent to the time “smelling” the object, that is, olfactory exploration. To date sniffing has not been used as a key marker of this behavior. Using our Noldus video tracking system combined with whole-body plethysmography we were able to address the question of whether the criteria used thus far to measure exploration, namely, distance and head angle to the object, are indeed related to enhanced sniff rates.  Figure 1 shows this to be the case: sniff rates are enhanced during periods that the WT mice were within 2 cm of the cotton tip and with their head aimed to it within 20° (OnFreq), as compared to other periods (OffFreq), for each trial type (PE3, S1, S2, and S3). For S1 trials this increased from 6.5 Hz to 9.5 Hz. PE3 shows low exploration time (OnTime < 1 sec) indicative of habituation, followed by a cross-habituation of ~14 sec exploration to the first social odor presentation (S1). This was followed by moderate, if apparently, inconsistent habituation during trials S2 and S3 (~8 sec). In contrast to the sniff rate being increased during proximity and orientation to the object, there was no evidence that sniff amplitudes were modulated by trial type.

**Figure 2:**
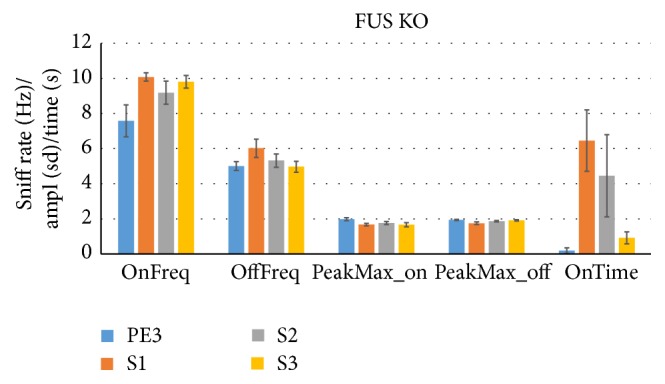
The sniff rate increases during cross-habituation in female FUS ko mice. The same as Figure 1, but now for FUS knock-out (ko) mice (*n* = 13), which have chronic oxidative stress and are a new model of accelerated aging. OnFreq and OffFreq: sniff rate (Hz); PeakMax_on and PeakMax_off: sniff peak amplitude (standard deviations of *z*-scored pressure signal). OnTime: odor object exploration time (s). All data are mean ± SEM.

Figure 2 shows that sniff rates followed the same pattern as WT mice, with S1 sniff rates at 10.1 Hz during proximity and orientation to the odorant, versus 6.0 Hz otherwise. (Note that the S1 exploration time was corrected from a rare (all data checked) Noldus output error (22.34 sec, mouse FUS627, set to 0 as the mouse was well over 2 cm away from the odorant)). We conclude that the proximity criterion of <2 cm and <20° to the odorant is indicative of olfactory exploration during habituation and cross-habituation for female mice of the 129 WT strain as well as for their FUS KO chronic oxidative stress counterparts.

**Figure 3:**
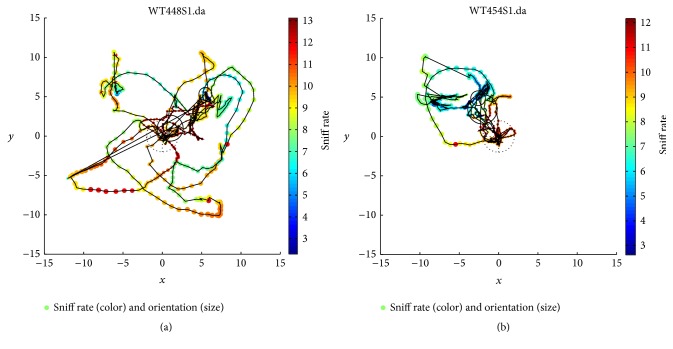
HaXha maps show sniffing rate related to proximity and orientation. Two S1 cross-habituation trials (first presentation of social odor) are represented by plotting the *x* and *y* nose location (circle), orientation (circle size), and sniff rate (circle color, see color bar legend, Hz) of each WT mouse during each 100 ms bin of the 120 sec trial. Subsequent bins are connected with lines. Centrally located (*x*, *y* = 0, 0) bulls-eye indicates the odor source location and 2 cm proximity criterion (2 cm radius). Large solid circle marks the position at trial start and the small circle the position at the end. Mice tended to sniff faster (more red circles) when closer to and oriented to (smaller circles) the odorant.

Figure 3 illustrates this finding for two WT mice during S1 trials by showing their path (large solid circle: start; small solid circle: end) in the cage (one dot per 100 ms bin, 1201 dots total), the head angle to the cotton tip (smaller size indicating smaller angle), and sniff rate (color coded). It is evident that sniff rates tend to be high and head angles low when they are near the centrally located odorant (dotted bulls-eye), though not exclusively so.

In the hypothetical absence of a relation between aforementioned criteria and sniff rates it would be untenable that such criteria measured olfactory exploration. In light of our findings that these criteria are predictive of sniff rate, the subtler question arises of whether the classical proximity and orientation criteria are optimal, either in isolation or combined, as a proxy for sniffing-mediated odorant exploration when sniff rate is not or cannot be measured (as is the general approach). We hence net explored this matter noncategorically (parametrically).

**Figure 4:**
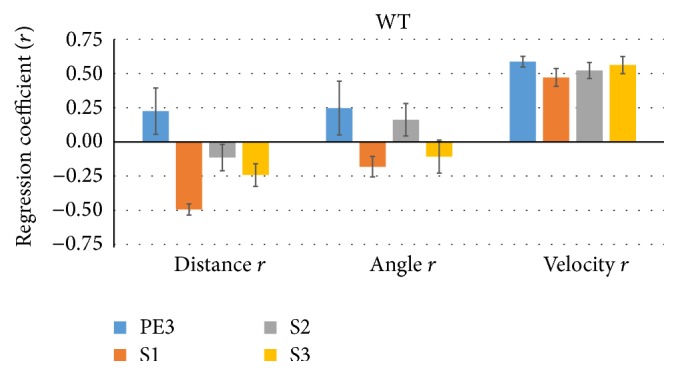
Proximity and velocity are correlated with S1 trial sniff rate in WT mice. On each trial (PE3, S3) for each WT mouse (*n* = 7) the mouse nose proximity (Distance *r*) and head orientation (Angle *r*) to the odor source and the velocity of the mice were regressed onto the sniff rate. Distance was negatively correlated during S1 trials, whereas velocity consistently positively correlated with sniff rate. The relation with angle was inconsistent.

**Figure 5:**
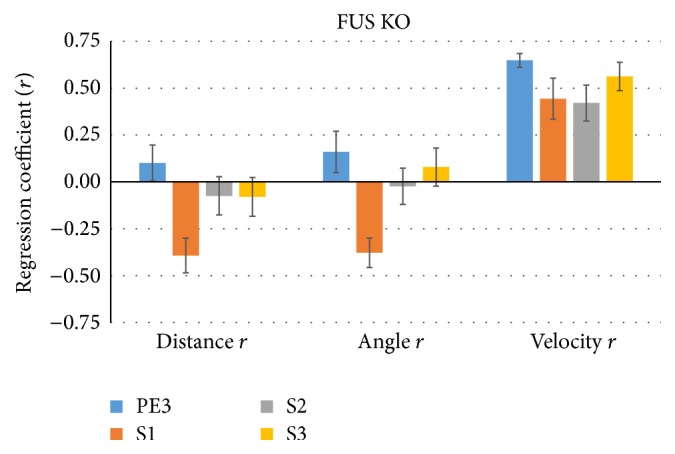
Proximity, orientation, and velocity are correlated with S1 trial sniff rate in FUS KO mice. The same as Figure 4, but for FUS KO mice (*n* = 13), showing similar results but with stronger negative correlation between head angle and sniff rate during S1 trials. Again, velocity consistently positively correlated with sniff rate.

Figure 4 shows the mean (±SEM, across 7 WT mice) linear regression coefficient between sniff rate and proximity (distance), orientation (angle), and the speed of WT mice's trajectory (velocity) across time bins. As expected from the prior results, distance is mostly negatively correlated, in particular during the cross-habituation S1 trials (*r* = −0.49) when sniff rate is modulated most strongly. However, the correlation between angle and sniff rate is quite inconsistent across trial types. Interestingly, the velocity of the mice was strongly and consistently positively correlated with sniff rate (*r* = 0.47–0.59; Figure 4, right). For FUS KO mice, shown in Figure 5, a similar set of relationships was evident, but now both angle and distance negatively correlated during S1 trials (*r* = −0.38).

**Figure 6:**
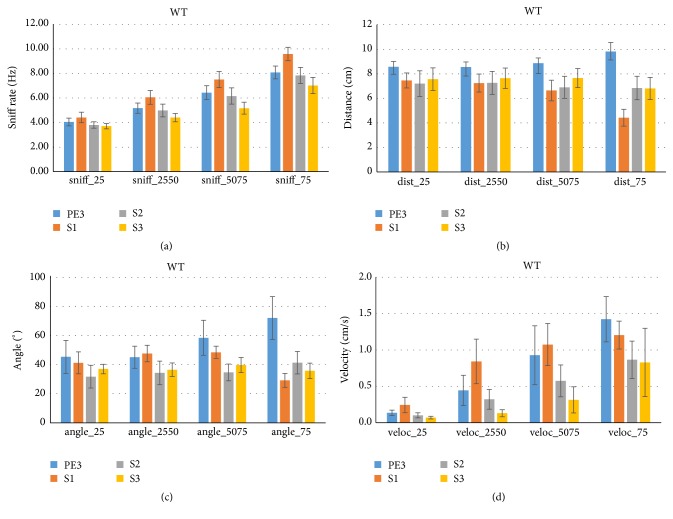
Sniff rate quartiles discriminate S1 proximity and consistently reveal WT mouse velocity. We explored how behavior varied as a function of sniff rate. For each trial type (PE3, S3) the 100 ms time bins were assigned to one of four quartiles of WT (*n* = 7) sniff rates (0th–25th, 25th–50th, 50th–75th, and 75th–100th percentile, (a)). Their associated nose distance (b) and head angle (c) to the odor source, as well as their velocity (d), were determined. Distance and to some extent head angle to the object were lower for the S1 trial during the high sniff rate top quartile time bins. Velocity consistently increased with increasing sniff rates across all trial types. FUS mice showed similar patterns (not shown).

In a complimentary approach we asked what behavioral parameters were associated with different sniff rates. Time bins were categorized to belong to one of four sniff rate quartiles (analyzed per trial) and associated distance, angle, and velocity were assessed.  Figure 6(a) shows the expected mean sniff rate (across bins, followed by across animals) increase from the 0–25th percentile of sniff rate (sniff_25), 25–50th percentile (sniff_2550), and 50–75th percentile (sniff_5075) to the 75–100th percentile (sniff_75). Whereas the lowest quartile shows similar sniff rates across trial types, at higher quartiles S1 sniff rates are highest. Exploration of the proximity during time bins associated with each sniff rate quartile shows that distance is smallest during S1 trials especially at the top quartile (Figure 6(b)). This indicates that small distance to the odorant during cross-habituation has a high specificity as a marker of olfactory exploration, a further validation of the proximity criterion. Although angle showed similar tendencies as distance, it lacked the specificity thereof (Figure 6(c)): orientation to the odor source occurred in overlapping degrees across sniff rate quartiles, notably S2 at lowest three quartiles.

Consistent with the strong and trial-type invariant positive correlation between velocity and sniff rate discussed before (Figures 4 and 5, right), we found that at higher sniff rate quartiles mice consistently showed higher velocities, albeit with different functions between trial types (Figure 6(d)). The near absence of movement at the lowest sniff quartile is rather striking. The same analysis for FUS KO mice (not shown) confirmed these WT findings, although with somewhat lower specificity of distance and higher specificity of angle for S1 trials than WT. Acceleration was also explored and found to be inconsistent across quartiles, trial types, and subject groups (not shown).

**Figure 7:**
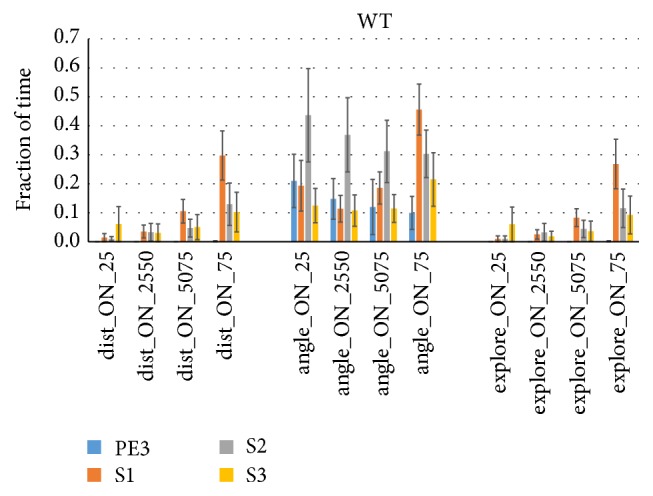
Sniff rate quartiles discriminate exploration time based on the proximity criterion. The same as Figure 6, but here the fraction of time the mouse spent sniffing at each quartile's sniff rate while fulfilling the <2 cm nose proximity criterion (left), the <20° head orientation criterion (middle) or both criteria was determined. WT mice (*n* = 7) were in close proximity to the odor for 30% of the time they sniffed fast (dist_ON_75) during S1 trials but 13% or less during other trial types (i.e., 87% or more of the time they sniffed at high rate they were >2 cm from the odorant during PE3, S2, or S3). For orientation the results were not as trial type specific or sniff rate specific (especially S2 at angle_ON_25: 44% of time oriented to odor during slow (lowest quartile) sniff rates). The orientation criterion does not appear to add to the exploration time fraction as determined by nose proximity (dist_ON), as explore_ON is quite similar to dist_ON. FUS KO mice showed similar results (not shown).

We further explored at what fraction of time when sniffing at each sniff rate quartile the WT mice would satisfy the categorical proximity (<2 cm), orientation (<20°), or their combined (<2 cm & <20°) criterion (Figure 7). It can be seen that during faster sniffing at the top quartile mice spent ~30% of their time in close proximity to the odorant (dist_ON_75) during S1 trials but much less time during other trials or at lower quartiles (<13%, Figure 7, left). During S1 trials mice spent 46% of time oriented to the odorant when sniffing fast (angle_ON_75) and less than 21% at lower sniff rates. However, during S2 trials they were similarly oriented to the odor source even at the lowest quartile. The combined criterion (explore_ON) largely follows that of the distance criterion alone. FUS KO data shows similar patterns, though with somewhat lower specificity for proximity and higher for orientation than WT shown here.

Thus far we have shown that complimentary analytical approaches confirm that proximity and (to a lesser degree) orientation to the odorant relate to enhanced sniffing, especially during S1 cross-habituation trials, lending credit to this long-used means of measuring odor object exploration duration (in the absence of a direct sniff rate measure). We also found that velocity may be an additionally useful factor to consider in quantifying odor exploration in the HaXha test, due to its strong and consistent relation to sniff rate.

**Figure 8:**
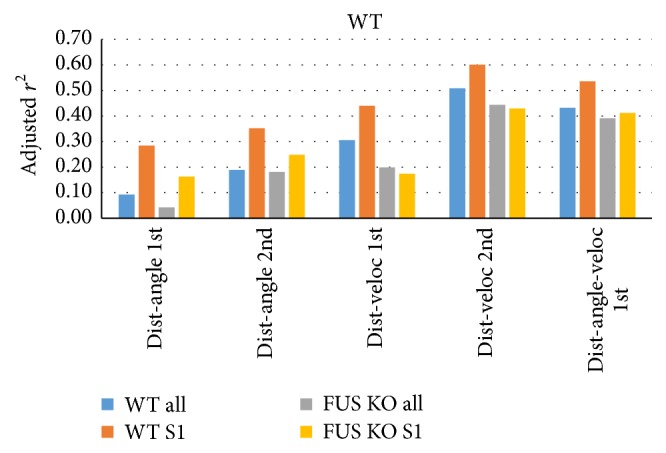
Distance and velocity combined predict sniff rates. Population vectors that include all time bins from all WT mice were used to regress both nose to odor source distance and head angle to odor source (dist-angle) or distance and velocity (dist-veloc) parametrically onto sniff rates using 1st- or 2nd-order equations (shown in Figure 9), for either all trials of WT or FUS KO mice or their S1 trials. The 2nd-order distance-velocity regression explained 43–60% of sniff rates (adjusted *r* ^2^; S1: *n* = 7 mice *∗* 1201 time bins = 8407 bins; all: *n* = 7 mice *∗* 1201 bins *∗* 4 trials = 33,628 time bins). A 3-way stepwise 1st-order multiple regression (dist-angle-veloc 1st, right) also robustly explained sniff rates.

**Figure 9:**
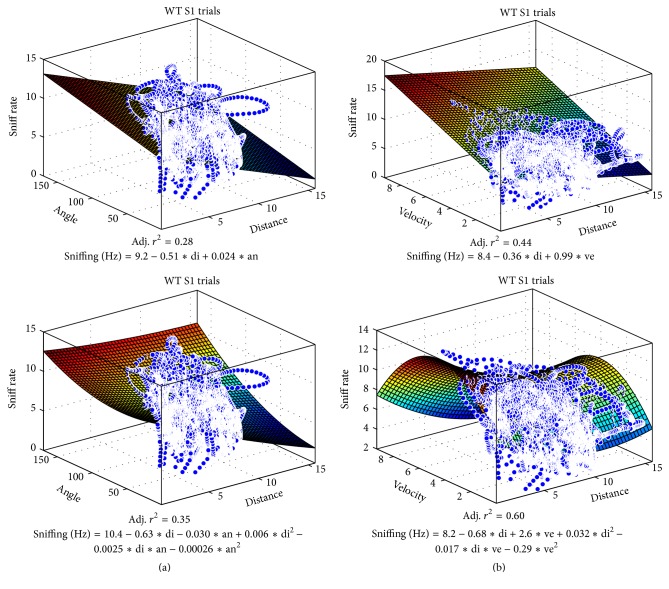
Distance and velocity combined predict sniff rates. Plots of the population vector sniff rates in state-space ((a) distance and angle; (b) velocity and distance) used in Figure 8 and the multiple regression equations and adjusted *r* ^2^ for all WT S1 trials combined (8407 time bins).

We therefore used multiple regression to see how well combinations of proximity, orientation, and velocity can predict sniff frequency parametrically (Figure 8). Population vectors were used to allow all time bins from all mice to be included in a single regression (each vector spanning 7 mice *∗* 1201 bins = 8407 time bins for S1 trials; 8407*∗*4 trials = 33,628 bins for all trials). While we found that distance and angle combined could explain 35% of WT S1 sniff rate variance (adjusted *r* ^2^, 2nd-order regression, dist-angle 2nd), distance and velocity explained a rather high 60% (dist-veloc 2nd) of WT S1 trial sniff rate variance, and >40% of sniff rate variance across all WT and FUS trials (WT all, FUS KO all). Distance and angle only explained up to 19% of sniff rate variance across all trials in both groups.  Figure 9 shows the respective sniff rate state-spaces for WT S1 trials and the regression equations ((a) angle and distance; (b) velocity and distance; top: 1st-order, bottom, 2nd-order). A stepwise multiple regression (*P* value to enter: 0.05) included all 3 factors and explained roughly as much as the 2-way 2nd-order velocity-distance regression (39–54%, Figure 8, right-most bars). These data suggest that the combined criterion of proximity and velocity may be more useful than proximity and orientation in estimating sniff-mediated odor exploration.

**Figure 10:**
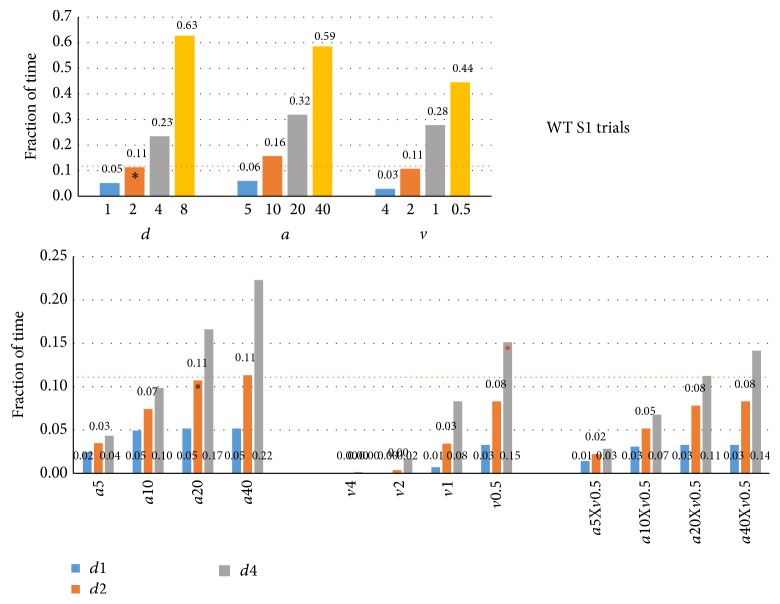
Exploration times at targeted criterion thresholds. Top: exploration time (as fraction of total trial time) was established for nose to odor source distance (*d*, <1, <2, <4, and <8 cm), head angle to odor source (*a*, <5, <10, <20, and <40°), and velocity (*v*, >4, >2, >1, and >0.5 cm/s) criteria at four threshold levels using population vectors (top) for S1 trials in WT mice (*n* = 7). Marked (*∗*) is *d*2 (distance < 2 cm) as standard criterion reference, yielding 11% (13.2 sec) exploration time. Relaxed criteria yield higher exploration times. Bottom: the effects of head angle and velocity criteria, separately (*a*5–*a*40,* v*4–*v*0.5) or combined (*a*5–*a*40 combined with* v*0.5) on exploration time using a nose to odor source distance <1, <2, or <4 cm (*d*1–*d*4) as additional criterion in each graph. Marked (*∗*, black) is the combined criterion *a* < 20° and *d* < 2 cm, being the commonly used “standard” criterion, which did not restrict the exploration time over criterion *d* < 2 cm alone (*d*2, Top). Also marked (*∗*, red) is the combination *d* < 4 cm and *v* > 0.5 cm (*d*4-*v*0.5), suggested as useful new criterion (see Results and Figure 11) that yielded 15% exploration time.

**Figure 11:**
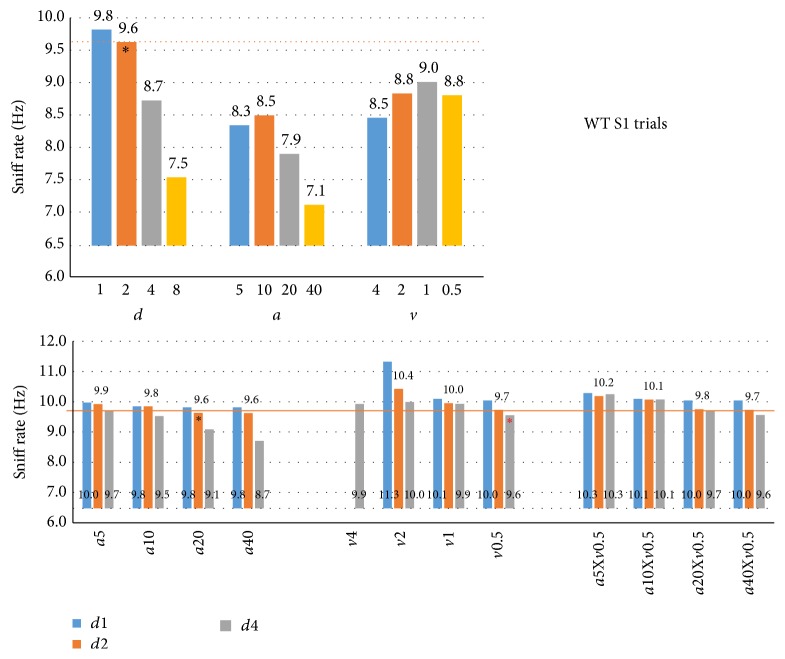
Sniffing rates at targeted criterion thresholds. Sniff rates associated with the same criteria as shown in Figure 10. Top: single criteria; Bottom: combined criteria. Relaxed criterion thresholds include bins with low sniff rates unrelated to odor source exploration. Stricter orientation criteria combined with distance <1 cm or <2 cm do not yield higher sniff rates during exploration (bottom left), but exploration time drops dramatically (Figure 10, bottom left). The relaxed proximity criterion (<4 cm) combined with velocity >0.5 cm/s (*∗*, red) yields sniff rates equivalent to the standard (*d*2-*a*10; 9.6 Hz; bottom center) and also increases exploration time from 11% to 15% (Figure 10), suggesting this to be a useful new HaXha odor exploration criterion. Similar results were found for other trial types and FUS KO mice (not shown).

In an effort to establish new criteria based on the above findings we used the S1 trial population vectors to see which exploration times (Figure 10) and exploration sniff rates (Figure 11) would result from using different criterion thresholds and their combinations. Optimal criteria should yield the highest exploration time without substantially lowering the sniff rate during it. Figure 10 (top) shows the fraction of time the mice would be considered to be exploring with distance criteria of less than 1, 2, 4, or 8 cm (left,* d*1–*d*8), angle of less than 5, 10, 20, or 40° (middle,* a*5–*a*40), or velocity of more than 4, 2, 1, or 0.5 cm/s (right,* v*4–*v*0.5). These specific thresholds were chosen to yield roughly parallel levels of exploration. The distance threshold <2 cm (*d*2) was marked, including a horizontal line for reference, as it is the standard criterion and yields exploration for 11% of time (13.2 sec for a 120 sec trial, like the trial-based Noldus result of 14.1 sec in Figure 1, right). Figure 10 (bottom) shows the fraction of time explored for their combinations at 3 distance thresholds. It can, for example, be seen that combining* d*2 with* a*20 does not substantially alter exploration time (11%), suggesting that these thresholds largely overlap over time, calling into question the usefulness of adding orientation to the* d*2 criterion.

Figure 11 (top) shows that no single threshold can substantially improve on* d*2, yielding 9.6 Hz sniffing during the 11% exploration time (close to 9.5 Hz OnFreq in Figure 1; 6.5 Hz is shown as *y*-axis bar cut-off as this is the OffFreq baseline sniff rate, i.e., when not exploring). Whereas the strictest angle thresholds yield peak rates of ~8.5 Hz, sniff rates decrease somewhat with stricter velocity thresholds (from 9.0 to 8.5 Hz). Sniff rate would increase slightly by tightening the distance threshold from <2 to <1 cm from odor source, but exploration time would drop dramatically from 11% to 5% (Figure 10, top:* d*2 versus* d*1).

Nearly all the combined criteria (Figure 11, bottom) show sniff rates similar or somewhat higher than when applying the* d*2 criterion (orange line). At* d*2 the various angle thresholds yield similar rates of 9.6–9.9 Hz (Figure 11, bottom left). Relaxing the distance threshold to <4 cm requires an angle threshold <10 to retain such rates but reduces exploration time from 11% to 7% (Figure 10, bottom left). Combining proximity with velocity (Figure 11, middle) shows that it can yield highest exploration sniff rates (*d*1 and* v*2; 11.3 Hz) but identifies only 0.02% as exploration. In contrast, relaxed distance threshold <4 cm and velocity >0.5 cm/s (*d*4 and* v*0.5, marked) yielded the same 9.6 Hz as the standard but* increased* exploration time from 11% to 15%, thereby being suggestive of a criterion better able to identify sniff-mediated odor exploration. Adding orientation to this combination did not improve matters (Figure 11, right).

**Figure 12:**
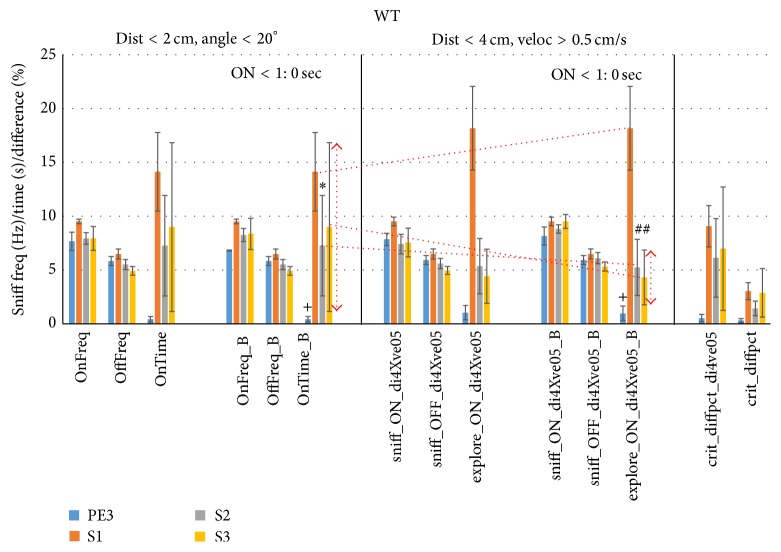
New combined proximity-velocity criterion improves HaXha exploration scoring in WT mice. To test the usefulness of the exploration criterion of the velocity >0.5 cm/s and distance <4 cm, the trials were scored accordingly (center) for sniff rate (Hz) when exploring (sniff_ON_di4Xve05) or not (sniff_OFF_di4Xve05) and exploration time (s; explore_ON_di4Xve05). The results of the standard criterion (*d* < 2 cm and angle < 20°) are shown on the left (same as Figure 1, left). Data were more robust upon removing sniff rates of mice with exploration time <1 sec (_B extension). The new criterion increased S1 exploration time (orange line) and decreased S2 and S3 exploration time variance (arrows), while retaining high sniff rates indicative of sniff-guided odorant exploration, leading to stronger statistical conclusions. The difference (%) in time bins meeting these two criteria was also assessed (% overlap; crit_diffpct_div4ve05) as well as between the Noldus distance-angle exploration marker output and the thresholded smoothed distance-angle score (crit_diffpct). ^*∗*^ *P* < 0.05; ^+^ *P* < 0.01; ^#^ *P* < 0.001 (paired 1-sided *t*-test versus S1).

**Figure 13:**
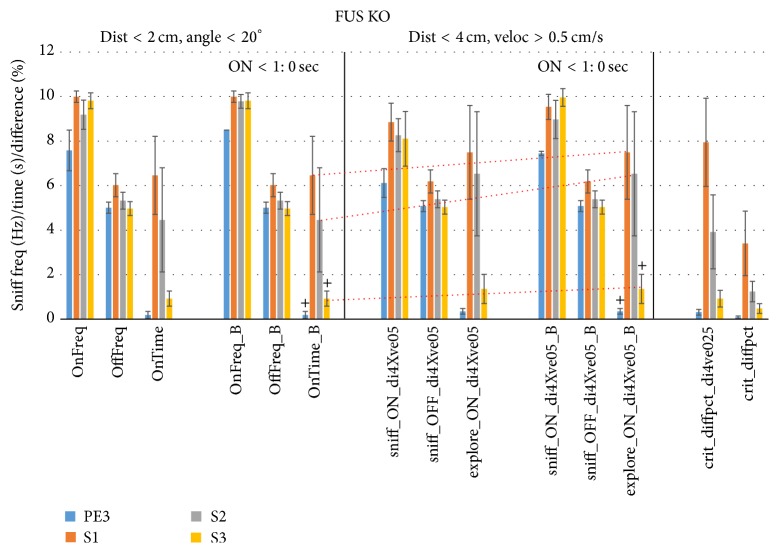
New combined proximity-velocity criterion improves HaXha exploration scoring in FUS KO mice. The same as Figure 12, but for FUS KO mice (*n* = 13). The new criterion increased S1, S2, and S3 exploration time (orange lines), while retaining high sniff rates indicative of sniff-guided odorant exploration. ^+^ *P* < 0.01 (paired 1-sided *t*-test versus S1).

We next tested this new criterion of distance threshold <4 cm and velocity >0.5 cm/s (*d*4-*v*0.5) to individual trials of WT (Figure 12, middle) and FUS KO mice (Figure 13, middle). The left graph in these figures (OnFreq, OffFreq, and OnTime) is identical to that of Figures 1 and 2 using the Noldus *d* < 2 cm and *a* < 20° criterion (using unsmoothed data) and is shown for reference. The neighboring graphs (marked “_B”) show these results upon removal of trials of mice yielding <1 sec exploration time, which helped robustness in particular for the* d*4-*v*0.5 criterion by raising S2 and S3 sniff rates during exploration and reducing their SEM (sniff_ON_di4Xve05_B) for both WT and FUS KO mice. The* d*4-*v*0.5 criterion increased WT PE nonexploratory sniff rates slightly but consistently (Sniff_OFF_di4Xve05_B) over the standard* d*2-*a*20 criterion (OffFreq_B, *P* < 0.001, paired 2-sided *t*-test, Figure 12) and also consistently, if slightly, increased FUS KO PE3, S2, and S3 nonexploratory sniff rates (*P* < 0.001, *P* < 0.05, and *P* < 0.001, resp., paired 2-sided *t*-test, Figure 13). No difference was found for sniff rates during exploration.

The* d*4-*v*0.5 criterion increased WT S1 trial exploration time (explore_ON_di4Xve05_B) over the standard* d*2-*a*20 criterion (OnTime_B) by 39% from 14.1 sec to 18.2 sec (orange line), without decreasing sniff rate (9.5 Hz, Figure 12). Meanwhile, S2 and S3 exploration times of WT mice decreased together with a larger reduction in SEM. This high SEM using* d*2-*a*20 was mostly due to a single outlying mouse (WT517) yielding 33 and 56 sec of exploration time for S2 and S3, respectively (data was verified for correct assessment). The new criterion is apparently more robust in avoiding such pitfalls, which can allow for a statistically more powerful (discriminating) assessment of habituation and cross-habituation. Indeed, whereas habituation was weak for S2 (*P* = 0.04, paired 1-sided *t*-test) and was not significant for S3 using the* d*2-*a*20 criterion, it was highly significant for S2 and S3 (*P* < 0.001) using the* d*4-*v*0.5 criterion. Other outcomes were roughly similar between the two criteria and for FUS KO mice (Figure 13) statistical conclusions based on exploration time did not differ between the two criteria.

The difference in time bins meeting these two criteria was also assessed (% overlap) and was ~6–9% for S1–S3 (crit_diffpct_div4ve05, Figure 12). The difference between the Noldus unsmoothed* d*2-*a*20 exploration time (USB-based acquired) output and the post hoc smoothed* d*2-*a*20 exploration time output was <3% (crit_diffpct). The FUS KO criterion comparison yielded similar results as for WT (Figure 13). S1 exploration time increased by 15% (6.5 sec to 7.5 sec), S2 by 44% (4.5 sec to 6.5 sec), and S3 by 0.5 sec (0.9 to 1.4 sec), without a concomitant increase in variance or reduction in associated sniff rate. Exploratory time bins also differed to a degree similar to WT (Figure 13, right).

**Figure 14:**
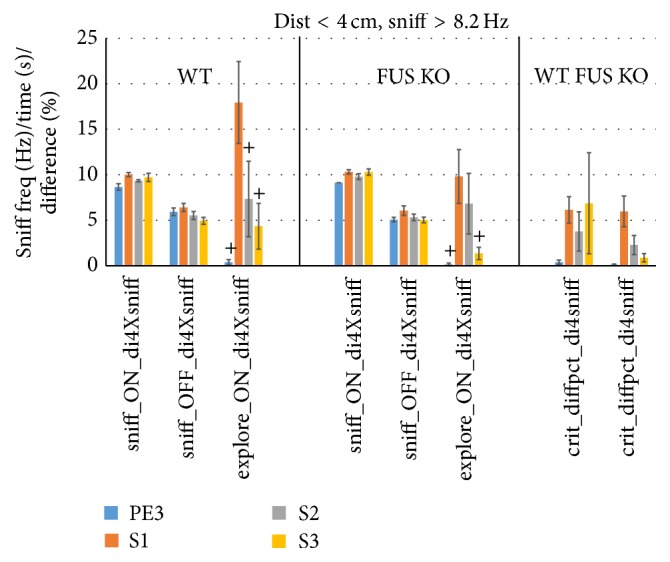
New combined proximity-sniffing criterion improves HaXha exploration scoring in mice. To demonstrate the usefulness of using sniff rate itself as an exploration criterion for WT (left, *n* = 7) and FUS KO (right, *n* = 13) mice, bins were scored using sniff rate >8.2 Hz (the 97.5th percentile of sniff_OFFdi4Xsniff, i.e., unusually high sniff rates when mice are not exploring) and distance <4 cm. Sniff rate (Hz) when exploring (sniff_ON_di4Xsniff) and not exploring (sniff_OFF_di4Xsniff) and exploration time (s; explore_ON_di4Xsniff) are shown. The difference (%) in time bins meeting this new and the standard criterion was also assessed (% overlap; crit_diffpct_div4sniff). ^+^ *P* < 0.01 (paired 1-sided *t*-test versus S1).

As we propose that exploration of an odor object should ultimately be guided by both proximity and the direct means of exploring it via sniffing, we lastly also show the scores using the criterion of distance <2 cm and sniff rate >8.2 Hz (Figure 14). This sniff rate threshold was chosen so as to be highly unusual when not exploring. It was calculated from the mean + 1.96 SD rate (97.5th percentile) using di < 4 cm and ve > 0.5 cm/s (Sniff_OFF_di4Xve05_B, Figures 12 and 13) and was the same for both groups of mice. The results were very similar to the results using the di < 4 cm and ve > 0.5 cm/s criterion.

## 4. Discussion

The present work explores the criteria used to score odor exploration time during the HaXha task, using sniff rate as the ultimate guide. Thorough analysis was afforded through the combined use of video tracking (Noldus) and whole-body plethysmography (Buxco). Although it could be recommended to measure sniff rate during the HaXha task to aid determination of exploration time in general, the sniff measure may typically not be available for various reasons. We hence sought to corroborate the validity of the criteria used thus far in the literature and explored if improvements were possible in absence of sniffing data in a total of 19 mice across 4 trial types.

We found that the use of <2 cm proximity to the odorant alone, or in combination with the somewhat redundant head orientation criterion of <20°, provided a remarkably accurate estimate of the odor exploration time. Sniff rates were clearly elevated during such exploration times (Figures 1 and 2), and distance and angle were correlated to sniff rate (Figures 4 and 5), depended on trial type and sniff rate (Figures 6 and 7) and together explained 35% of sniff rate during S1 trials (Figures 8 and 9). The exploration sniff rate using the proximity criterion alone or the combined proximity-orientation criterion also could not be significantly improved upon without a drastic loss of identified exploration time (Figures 10 and 11). We conclude that the commonly used proximity criterion (<2 cm) alone or combined with head angle (<20°) is a very effective tool to estimate odor exploration in the HaXha test.

We also explored the use of other behavioral variables to estimate odor exploration time. Whereas the acceleration of mice was found to be an inconsistent predictor (not shown), velocity was remarkably consistent (Figure 4) across trial types and subjects. Velocity was strikingly related to sniff rate, albeit with different functions across trial types (Figure 6), suggesting that sniffing and moving are somehow coupled and in a context-dependent way. Proximity and velocity combined explained 60% of sniff rate during S1 trials, double that of the standard criterion (Figures 8 and 9). Although using distance and velocity as criterion was unable to appreciably increase sniff rate during exploration time over the standard without massively reducing the exploration time, using velocity (>0.5 cm) combined with a relaxed distance criterion (<4 cm) retained the high sniff rate while increasing S1 exploration time (Figures 10 and 11). When tested on a trial-trial basis the new criterion typically increased exploration time by 15–44%, and was more robust by avoiding outliers leading to substantially different and better statistical conclusions in case of WT mice (Figures 12 and 13). For 129 strain mice we can hence recommend the criterion of proximity <2 cm and velocity >0.5 cm/s over the standard criterion of proximity <2 cm and head angle <20° when video tracking is available. We plan to scrutinize this for other mouse strains in the near future.

It is clear that mice olfactorily explore the entire HaXha environment as indicated by increased sniff rate when traveling through the box (Figure 3) and sniff fast only 30% of the time near the odorant even during S1 trials (Figure 7). Furthermore, while velocity appeared strongly related to sniff rate in general, at very high velocities (>4 cm/s) it was only apparent when very near the odor source (Figure 11,* d*1*v*2). When further away from the source higher velocities appeared to coincide with reduced sniff rates (e.g., see widely spaced circles in Figure 3) evidenced by the saddle form of the velocity-distance regression in Figure 9. The general relationship between velocity and sniffing was somewhat surprising to us as we expected the mice to also show “stop and sniff” behavior which we did not find evidence for given the effectiveness of the velocity threshold criterion. It hence appears that 129 mice modulate their body position at least somewhat while sniffing the cotton tip. It should be pointed out that, unlike common setups where the tip is mounted on the cage lid sufficiently elevated from the floor to induce rearing and concomitant low velocity once reared, our cotton tip was mounted 1 cm from the floor so as not to require rearing.

While not being the focus of this paper, we found that FUS KO mice showed reduced habituation and cross-habituation compared to WT mice (di4-ve05). Both groups showed significant cross-habituation (PE3 versus S1), but WT mice explored the S1 odorant for longer duration (18.2 ± 3.9 sec, Figure 12, center) than FUS KO mice (7.5 ± 2.1 sec, Figure 13, center). This difference was quite significant using the new di4-ve05 exploration criterion (*P* = 0.019, 1-sided unpaired *t*-test) and using the di2-an20 criterion (*P* = 0.046, 1-sided unpaired *t*-test). Sniff rates did not differ between the groups. WT mice significantly cross-habituated during the S2 trial (5.2 ± 2.6 sec), whereas the FUS KO mice did not (6.5 ± 2.8 sec). These findings suggest that FUS KO mice show a mild olfactory discriminatory deficit. This conclusion would not have been reached using the standard suboptimal criteria.

The complete HaXha sessions consisted of 3 presentations each of mineral oil (MO, diluent control), followed by amyl acetate (AA), PE, and finally S. We intentionally omitted stimulus presentations prior to PE3 from this paper for sake of clarity of an already complex data set. The chosen trials were deemed sufficient to demonstrate large differences in exploration time accompanied by similarly large differences in sniff rates. For this entire series (MO, AA, PE, and S) repeated measures ANOVA showed a significant effect of trial on exploration time for WT mice (*P* < 0.01, *F*_2.3,30.0_ = 5.8) and for FUS KO mice (*P* < 0.001, *F*_3.0,62.0_ = 11.7) when using the standard Noldus criteria. WT mice only showed significant cross-habituation between PE3 and S1 and no significant habituation. FUS mice showed significant cross-habituation (increase) between AA3 and PE1 (*P* < 0.05) and PE3 and S1 (*P* < 0.001) and significant habituation (decrease from first to third trial) for MO and PE (*P* < 0.05) and S (*P* < 0.001; all Bonferroni-corrected post hoc *t*-test on exploration time based on standard criteria). These results support the notion that the FUS mice displayed haXha, whereas this is less evident for the WT mice (due to absent habituation) in which case differences in interest in PE and S may be responsible for the different exploration times.

As proof of principle we also showed that the criterion based on both sniff rate and proximity is sensitive and robust (Figure 14). It yielded remarkably similar results to the other indirect approaches to assess olfactory guided exploration, again attesting to their effectiveness. The most notable difference was the higher FUS KO exploration time from 6.5 ± 1.8 (di2an20) and 7.5 ± 2.1 (di4ve05) to 9.8 ± 2.9 sec, (di4sniff8.2) which subsequently was marginally significantly lower than that of WT (17.9 ± 4.5 sec, *P* = 0.076, 1-sided unpaired *t*-test). Other sniff thresholds may show somewhat different results, but preliminary exploration suggests that the outcome is rather robust to sniff rate threshold (not shown).

In conclusion, we confirm that standard HaXha exploration criteria are fairly accurate at assessing sniff-modulated odorant exploration. We suggest that using velocity or sniffing itself rather than head orientation, combined with proximity, as criterion provides more accurate and more robust results. We further suggest that combined use of video tracking and sniff measurements is optimally suited to perform HaXha experiments.

## References

[1] Thompson R. F., Spencer W. A. (1966). Habituation: a model phenomenon for the study of neuronal substrates of behavior. *Psychological Review*.

[2] Rankin C. H., Abrams T., Barry R. J. (2009). Habituation revisited: an updated and revised description of the behavioral characteristics of habituation. *Neurobiology of Learning and Memory*.

[3] Sundberg H., Døving K., Novikov S., Ursin H. (1982). A method for studying responses and habituation to odors in rats. *Behavioral and Neural Biology*.

[4] Beauchamp G. K., Wellington J. L. (1984). Habituation to individual odors occurs following brief, widely-spaced presentations. *Physiology and Behavior*.

[5] Brown R. E. (1988). Individual odors of rats are discriminable independently of changes in gonadal hormone levels. *Physiology and Behavior*.

[6] Deiss V., Féron C., Baudoin C. (1999). Discrimination of olfactory sexual cues in staggerer mutant male mice. *Physiology and Behavior*.

[7] Wilson D. A. (2000). Odor specificity of habituation in the rat anterior piriform cortex. *Journal of Neurophysiology*.

[8] Cleland T. A., Morse A., Yue E. L., Linster C. (2002). Behavioral models of odor similarity. *Behavioral Neuroscience*.

[9] Linster C., Johnson B. A., Morse A., Yue E., Leon M. (2002). Spontaneous versus reinforced olfactory discriminations. *The Journal of Neuroscience*.

[10] McNamara A. M., Magidson P. D., Linster C., Wilson D. A., Cleland T. A. (2008). Distinct neural mechanisms mediate olfactory memory formation at different timescales. *Learning and Memory*.

[11] Wrenn C. C., Harris A. P., Saavedra M. C., Crawley J. N. (2003). Social transmission of food preference in mice: methodology and application to galanin-overexpressing transgenic mice. *Behavioral Neuroscience*.

[12] Mihalick S. M., Langlois J. C., Krienke J. D., Dube W. V. (2000). An olfactory discrimination procedure for mice. *Journal of the Experimental Analysis of Behavior*.

[13] Yang M., Crawley J. N. (2009). Simple behavioral assessment of mouse olfaction. *Current Protocols in Neuroscience*.

[14] Slotnick B., Coppola D. M. (2015). Odor-cued taste avoidance: a simple and robust test of mouse olfaction. *Chemical Senses*.

[15] Garrido M., Terrón M. P., Rodríguez A. B. (2013). Chrononutrition against oxidative stress in aging. *Oxidative Medicine and Cellular Longevity*.

[16] Wesson D. W., Verhagen J. V., Wachowiak M. (2009). Why sniff fast? The relationship between sniff frequency, odor discrimination, and receptor neuron activation in the rat. *Journal of Neurophysiology*.

[17] Wesson D. W., Donahou T. N., Johnson M. O., Wachowiak M. (2008). Sniffing behavior of mice during performance in odor-guided tasks. *Chemical Senses*.

[18] Verhagen J. V., Wesson D. W., Netoff T. I., White J. A., Wachowiak M. (2007). Sniffing controls an adaptive filter of sensory input to the olfactory bulb. *Nature Neuroscience*.

[19] Wesson D. W., Carey R. M., Verhagen J. V., Wachowiak M. (2008). Rapid encoding and perception of novel odors in the rat. *PLoS Biology*.

[20] Averback P. (1983). Two new lesions in Alzheimer's disease. *The Lancet*.

[21] Esiri M. M., Wilcock G. K. (1984). The olfactory bulbs in Alzheimer's disease. *Journal of Neurology Neurosurgery and Psychiatry*.

[22] Simpson J., Yates C. M., Gordon A., St Clair D. M. (1984). Olfactory tubercle choline acetyltransferase activity in Alzheimer-type dementia, Down's syndrome and Huntington's chorea. *Journal of Neurology, Neurosurgery, and Psychiatry*.

[23] Warner M. D., Peabody C. A., Flattery J. J., Tinklenberg J. R. (1986). Olfactory deficits and Alzheimer's disease. *Biological Psychiatry*.

[24] Mrochen A., Marxreiter F., Kohl Z. (2016). From sweet to sweat: hedonic olfactory range is impaired in Parkinson's disease. *Parkinsonism and Related Disorders*.

[25] Cavaco S., Gonçalves A., Mendes A. (2015). Abnormal olfaction in Parkinson's disease is related to faster disease progression. *Behavioural Neurology*.

[26] Caminiti F., De Salvo S., De Cola M. C. (2014). Detection of olfactory dysfunction using olfactory event related potentials in young patients with multiple sclerosis. *PLoS ONE*.

[27] Rolet A., Magnin E., Millot J. L. (2013). Olfactory dysfunction in multiple sclerosis: evidence of a decrease in different aspects of olfactory function. *European Neurology*.

[28] de Haro-Licer J., Roura-Moreno J., Vizitiu A., González-Fernández A., González-Ares J. A. (2013). Long term serious olfactory loss in colds and/or flu. *Acta Otorrinolaringologica Espanola*.

[29] Landis B. N., Vodicka J., Hummel T. (2010). Olfactory dysfunction following herpetic meningoencephalitis. *Journal of Neurology*.

[30] Lötsch J., Reither N., Bogdanov V. (2015). A brain-lesion pattern based algorithm for the diagnosis of posttraumatic olfactory loss. *Rhinology*.

[31] Doty R. L., Kamath V. (2014). The influences of age on olfaction: a review. *Frontiers in Psychology*.

[32] Coronas-Sámano G., Portillo W., Beltrán Campos V., Medina-Aguirre G. I., Paredes R. G., Diaz-Cintra S. (2014). Deficits in odor-guided behaviors in the transgenic 3xTg-AD female mouse model of Alzheimer's disease. *Brain Research*.

[33] Wesson D. W., Levy E., Nixon R. A., Wilson D. A. (2010). Olfactory dysfunction correlates with amyloid-*β* burden in an Alzheimer's disease mouse model. *The Journal of Neuroscience*.

[34] Yazlovitskaya E. M., Voziyan P. A., Manavalan T., Yarbrough W. G., Ivanova A. V. (2015). Cellular oxidative stress response mediates radiosensitivity in Fus1-deficient mice. *Cell Death and Disease*.

[35] Yazlovitskaya E. M., Uzhachenko R., Voziyan P. A., Yarbrough W. G., Ivanova A. V. (2013). A novel radioprotective function for the mitochondrial tumor suppressor protein Fus1. *Cell Death & Disease*.

